# Protein Kinase A and High-Osmolarity Glycerol Response Pathways Cooperatively Control Cell Wall Carbohydrate Mobilization in *Aspergillus fumigatus*

**DOI:** 10.1128/mBio.01952-18

**Published:** 2018-12-11

**Authors:** Leandro José de Assis, Adriana Manfiolli, Eliciane Mattos, João H. T. Marilhano Fabri, Iran Malavazi, Ilse D. Jacobsen, Matthias Brock, Robert A. Cramer, Arsa Thammahong, Daisuke Hagiwara, Laure Nicolas Annick Ries, Gustavo Henrique Goldman

**Affiliations:** a Faculdade de Ciências Farmacêuticas de Ribeirão Preto, Universidade de São Paulo, São Paulo, Brazil; b Departamento de Genética e Evolução, Centro de Ciências Biológicas e da Saúde, Universidade Federal de São Carlos, São Paulo, Brazil; c Research Group Microbial Immunology, Leibniz Institute for Natural Product Research and Infection Biology, Hans Knoell Institute, Jena, Germany; d Fungal Genetics and Biology Group, School of Life Sciences, University of Nottingham, Nottingham, United Kingdom; e Geisel School of Medicine at Dartmouth, Department of Microbiology and Immunology, Hanover, New Hampshire, USA; f Department of Microbiology, Faculty of Medicine, Chulalongkorn University, Bangkok, Thailand; g Faculty of Life and Environmental Sciences, University of Tsukuba, Ibaraki, Japan; h Faculdade de Medicina de Ribeirão Preto, Universidade de São Paulo, São Paulo, Brazil; Karlsruhe Institute of Technology (KIT); University of Wisconsin-Madison; Yonsei University

**Keywords:** *Aspergillus fumigatus*, cell wall, glycogen, high-osmotic glycerol pathway, protein kinase A, trehalose, SakA, MpkC

## Abstract

Aspergillus fumigatus mitogen-activated protein kinases (MAPKs) are involved in maintaining the normal morphology of the cell wall and providing resistance against cell wall-damaging agents. Upon cell wall stress, cell wall-related sugars need to be synthesized from carbohydrate storage compounds. Here we show that this process is dependent on cAMP-dependent protein kinase A (PKA) activity and regulated by the high-osmolarity glycerol response (HOG) MAPKs SakA and MpkC. These protein kinases are necessary for normal accumulation/degradation of trehalose and glycogen, and the lack of these genes reduces glucose uptake and glycogen synthesis. Alterations in glycogen synthesis were observed for the *sakA* and *mpkC* deletion mutants, which also displayed alterations in carbohydrate exposure on the cell wall. Carbohydrate mobilization is controlled by SakA interaction with PkaC1 and PkaR, suggesting a putative mechanism where the PkaR regulatory subunit leaves the complex and releases the SakA-PkaC1 complex for activation of enzymes involved in carbohydrate mobilization. This work reveals the communication between the HOG and PKA pathways for carbohydrate mobilization for cell wall construction.

## Abstract (precis)

Aspergillus fumigatus is an opportunistic human pathogen causing allergic reactions or systemic infections such as invasive pulmonary aspergillosis, especially in immunocompromised patients. The fungal cell wall is the main component responsible for recognition by the immune system, due to the specific composition of polysaccharide carbohydrates exposed on the surface of the fungal cell wall called pathogen-associated molecular patterns (PAMPs). Key enzymes in the fungal cell wall biosynthesis are a good target for fungal drug development. This report elucidates the cooperation between the HOG and PKA pathways in the mobilization of carbohydrates for fungal cell wall biosynthesis. We suggest that the reduced mobilization of simple sugars causes defects in the structure of the fungal cell wall. In summary, we propose that SakA is important for PKA activity, therefore regulating the availability and mobilization of monosaccharides for fungal cell wall biosynthesis during cell wall damage and the osmotic stress response.

## INTRODUCTION

The rise in opportunistic fungal infections has become a major concern for human health, especially in immunocompromised patients (1, 2). Fungal pathogens, such as Aspergillus fumigatus, Cryptococcus neoformans, and Candida albicans, have evolved several strategies to escape from the host immune defenses, including masking of cell wall-associated carbohydrates that constitute important fungal pathogen-associated molecular patterns (PAMPs) (3). PAMPs are recognized and bound by pattern recognition receptors (PRRs) present on the cell surface of host immune cells. Their activation results in immune response activation and the recruitment of mature neutrophils and macrophages, which promote degradation of fungal cells within mature phagosomal compartments and therefore clear the host of a potential infection threat (4).

The fungal cell wall is a complex structure, consisting of linear and branched polysaccharides and proteins that present the front line in host-pathogen interactions, protecting the fungus against host-associated stresses as well as immunomodulatory and evasive properties (5, 6). In addition, the cell wall structure is dynamically remodeled in a condition-dependent manner, resulting in changes to cell wall carbohydrate composition (7). The cell wall of A. fumigatus consists of linear and branched polysaccharides, notably α- and β-glucans, chitin, and galactomannans. Glucose is the basic unit of all types of glucans and also serves as a precursor for *N*-acetylglucosamine (NAG) in the chitin homopolymer. Galactose, which is a glucose epimer, is also the major monosaccharide in galactomannans (8). Glucose is therefore the predominant cell wall-associated monosaccharide, constituting more than 50% of all fungal cell wall polysaccharides (9–12). Glucose can be taken up from the extracellular environment and/or produced from gluconeogenesis and intracellular carbohydrate storage compounds such as trehalose and glycogen when required. The biosynthesis of fungal cell wall polysaccharides is achieved through a series of enzymatic steps that convert glucose to glucan, NAG, and galactomannan (8, 13–17). Deletion of A. fumigatus genes encoding enzymes that catalyze these steps results in several cell wall defects that impact pathogenesis, highlighting the importance of intracellular sugar metabolism for fungal cell wall function and virulence (6, 14, 18, 19).

Glucose utilization and trehalose and glycogen degradation are regulated by the protein kinase A (PKA) pathway (20–22). The presence of glucose is sensed by a heterotrimeric G-protein, with the Gα subunit activating adenylate cyclase, resulting in an increase in intracellular cAMP pools and subsequent PKA activation (23, 24). In eukaryotes, PKA is composed of a heterotetramer containing dimers of both a catalytic (PkaC1) and regulatory (PkaR) subunits. Binding of cAMP to PkaR results in the release of the active PkaC1 homodimer and subsequent downstream pathway regulation (24–26). Trehalose serves as an energy source during fungal germination at the first stages of development (27) and has been shown to be important for fungus-mediated infections (28–30). Deletion of the A. fumigatus *pkaC1* causes defects in conidium germination and fungal cell wall organization and attenuates virulence in a mouse model of infection (31–34). Similarly, glycogen breakdown and trehalose synthesis have been shown to be important for Magnaporthe oryzae plant infection (28, 30). In addition, A. fumigatus glycogen synthase kinase has been proposed to be a target for treating invasive aspergillosis as it is important for Aspergillus growth (35).

The high-osmolarity glycerol (HOG) pathway comprises the sequential stimulation of a mitogen-activated protein kinase (MAPK) cascade that is triggered upon osmotic, cell wall, and oxidative stresses resulting in the activation of the effector kinases SakA and MpkC (36). These protein kinases can then translocate to the nucleus and/or regulate the activity of additional protein targets, thereby modulating a cellular response to the extracellular environment (36). The Δ*sakA* and the double Δ*mpkC* Δ*sakA* mutants were more sensitive to osmotic and oxidative stresses and to cell wall-damaging agents. This work aimed to understand why the A. fumigatus HOG pathway is important for the activation of the cell wall integrity (CWI) pathway. Importantly, cross talk between the PKA pathway and the CWI and HOG pathways has been observed in several fungi (37, 38), although the mechanistic details of these interactions remain unknown in A. fumigatus. We hypothesize that there is a link between the PKA, CWI, and HOG pathways, which are important for the mobilization of storage sugars, such as trehalose and glycogen, and cell wall remodeling during osmotic and cell wall stresses. Our work aims to investigate a possible cooperation between the HOG and PKA pathways for carbohydrate mobilization for cell wall construction.

## RESULTS

### Carbohydrate mobilization is impaired in the Δ*sakA,* Δ*mpkC*, and Δ*sakA* Δ*mpkC* mutants.

**FIG 1:**
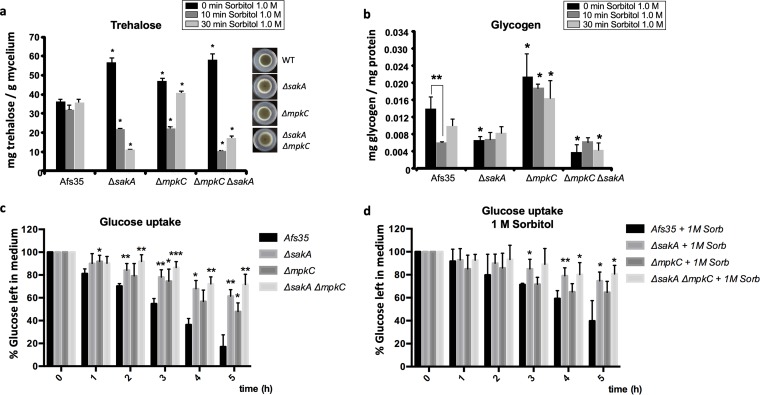
The role of the HOG MAPKs in controlling trehalose, glycogen, and glucose uptake levels. Strains were grown in complete medium for 20 h before 1 M sorbitol was added, and intracellular (a) trehalose levels and (b) glycogen content were measured. Radial growth of the strains on minimal medium is also shown in panel a. Strains were grown in complete medium for 20 h and then transferred to minimal medium supplemented with 1% glucose for 5 h, and (c) glucose uptake, represented by the percentage of residual glucose in the supernatant, was measured in the absence or (d) presence of 1 M sorbitol. Standard deviations represent the average from 3 biological replicates. Statistical differences were calculated using a paired *t* test (*, *P* < 0.05; **, *P* < 0.01; and ***, *P* < 0.001) based on comparison with the WT strain at the same time point.

A previous study (39) indicated that the MAPKs SakA and MpkC are important for trehalose and glycogen carbohydrate mobilization during 1 M sorbitol-induced osmotic stress, focusing our attention on HOG pathway activation by osmotic stress instead of oxidative and high-temperature stresses. High-throughput RNA sequencing (RNA-seq) showed a clear downregulation of genes encoding enzymes required for glycogen and trehalose metabolism, as well as genes encoding protein kinases (cAMP-dependent protein kinases and glucokinase) known to be involved in carbon source signaling in the Δ*sakA*, Δ*mpkC*, and Δ*sakA* Δ*mpkC* strains (39) (see Table S1 in the supplemental material). To further characterize carbohydrate mobilization during osmotic stress and to confirm the RNA-seq data, intracellular trehalose and glycogen levels were measured in the Δ*sakA*, Δ*mpkC*, and Δ*sakA* Δ*mpkC* strains when grown for 24 h in complete medium and after the addition of 1 M sorbitol for 10 min and 30 min (Fig. 1a and b). In the wild-type (WT) strain, trehalose concentrations remained the same under all conditions (Fig. 1a). In contrast, trehalose levels were significantly higher in the deletion strains under unstressed conditions, whereas osmotic stress caused a significant reduction in trehalose levels compared to the WT strain (Fig. 1a). The WT strain primarily consumed glycogen during osmotic stress, especially during the first 10 min of exposure to sorbitol (Fig. 1b), suggesting that glycogen, instead of trehalose, is used to counteract osmotic stress encountered by the cells. In the Δ*sakA* and Δ*sakA* Δ*mpkC* strains, however, glycogen levels were significantly reduced, whereas the Δ*mpkC* strain had increased intracellular glycogen concentrations under all conditions compared to the WT strain (Fig. 1b).

10.1128/mBio.01952-18.3 TABLE S1 Summary of the RNA-seq data based on Pereira Silva et al. (39). Download Table S1, XLSX file, 0.5 MB. Copyright © 2018 de Assis et al. 2018 de Assis et al. This content is distributed under the terms of the Creative Commons Attribution 4.0 International license.

To determine whether the alterations in trehalose and glycogen levels observed above are related to glucose uptake and/or signaling defects in the MAPK deletion mutants, the glucose concentration in the supernatant was first measured when strains were grown in complete medium for 20 h and after transfer to minimal medium (MM) supplemented without (Fig. 1c) or with (Fig. 1d) 1 M sorbitol for 5 h. Glucose uptake was severely reduced in the Δ*sakA*, Δ*mpkC*, and Δ*sakA* Δ*mpkC* strains in the presence and absence of osmotic stress, suggesting that these strains are not able to transport glucose like the wild-type strain, as observed in RNA-seq for expression of genes involved in carbohydrate transport (Table S1).

### PKA and the HOG MAPKs are crucial for cell wall structure.

**FIG 2:**
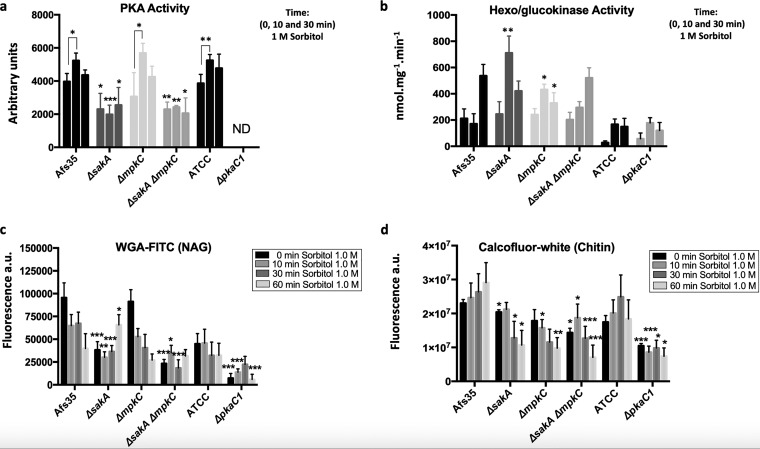
Requirement of HOG MAPK SakA for proper PKA activity and fungal cell wall content/organization. Strains were grown in complete medium for 20 h before 1 M sorbitol was added and (a**)** PKA activity was measured in the WT (wild-type) and Δ*sakA*, Δ*mpkC*, and Δ*sakA* Δ*mpkC* mutants during 10 and 30 min under sorbitol treatment. (b) Hexo/glucokinase activities were determined under the same conditions described in panel a. Cell surface (c) *N*-acetylglucosamine (NAG) and (d) chitin contents were measured when strains were grown in 200 μl liquid minimal medium for 16 h before 1 M sorbitol was added for the indicated time points. Mycelia were fixed with UV light before NAG was stained with WGA-FITC and chitin was stained with calcofluor white. Standard deviations represent the average from 3 biological replicates. Statistical differences were calculated using a paired *t* test (*, *P* < 0.05; **, *P* < 0.01; and ***, *P* < 0.001) based on comparison with the WT strain at the same time point or compared with the control of the same strain (line) for each time point.

The MAPKs of the HOG signaling pathway were shown to be involved in the transcriptional regulation of genes encoding regulatory and catalytic subunits of protein kinase A (PKA) (39), an enzyme required for cellular glucose signaling (Table S1). PKA activity was measured in the Δ*sakA*, Δ*mpkC*, and Δ*sakA* Δ*mpkC* strains when grown for 20 h in complete medium and after the addition of 1 M sorbitol for 10 and 30 min to probe for any defects in the glucose signaling pathway (Fig. 2a). In agreement with the RNA-seq data (Table S1), PKA activity was significantly reduced in the Δ*sakA* and Δ*sakA* Δ*mpkC* strains but not in the Δ*mpkC* strain under all conditions compared to the WT strain. These results suggest cooperation between the HOG and PKA pathways in A. fumigatus.

In Aspergillus nidulans, glucose uptake and hexo/glucokinase activities were impaired in the Δ*pkaA* strain (20). Consistently, the RNA-seq data showed a reduction in the expression of the glucokinase-encoding gene *glkA* in A. fumigatus in the MAPK deletion strains under osmotic stress conditions (39) (Table S1). In A. nidulans, phosphorylation of glucose by hexo/glucokinases during the first step of glycolysis was shown to be crucial for carbon catabolite repression (CCR) signaling (40). Indeed, hexo/glucokinase-mediated glucose phosphorylation during the first step of glycolysis is crucial for fungal cell wall construction as either single or double deletions of the corresponding genes resulted in strains with highly increased sensitivity to cell wall-damaging agents and sorbitol-mediated osmotic stress (see Fig. S1a to e in the supplemental material). Furthermore, the Δ*glkA* Δ*hxkA* double deletion mutant had attenuated virulence in both neutropenic and nonneutropenic mouse models of pulmonary aspergillosis (Fig. S1f to k). This emphasizes the importance of the glucose phosphorylation pool for further intracellular utilization of this monosaccharide and highlights the importance of cell wall synthesis/remodeling and growth for A. fumigatus pathogenesis.

10.1128/mBio.01952-18.2 FIG S1 Deletion of the regulatory enzymes in the carbohydrate mobilization. The Δ*hxkA*, Δ*glkA*, and Δ*hxkA* Δ*glkA* double mutant strains were grown on minimal medium supplemented with 1% Casamino acids and with increasing concentrations of (a) Congo red (CR), (b) calcofluor white (CFW), (c) sorbitol, and (d) NaCl for 48 h. Colony radial diameters were measured, and inhibition of growth is indicated as a percentage compared to the control condition. (Without cell wall-perturbing drug, growth is considered 100%.) Growth is depicted in the form of a heat map. Experiments were carried out in biological triplicates, and statistical differences were calculated using a paired *t* test (*, *P* < 0.05; **, *P* < 0.01; and ***, *P* < 0.001) based on comparison with the wild-type (WT) strain in the presence of the highest concentration of the tested compound. (e) Radial growth using MM supplemented with 1% sorbitol as a carbon source. Shown are survival curves of (f and g) mice immunocompromised with cortisone acetate and infected with A. fumigatus wild-type (WT), deletion mutants (Δ*glkA*, Δ*hxkA*, Δ*hxkA ΔglkA*), and complemented strains (cGlkA, cHxkA) at 2 × 10^5^ conidia per mouse. (i to k) Survival of mice using the same strains as shown in panels f and g with mice immunocompromised with cyclophosphamide (*n* = 10 per group). Deletion of the glycogen synthase-encoding gene *gsyA* does not result in hypersensitivity to cell wall stress but results in the loss of the caspofungin paradoxical effect. (l) The wild-type (WT) and Δ*gsyA* strains were grown in serial dilutions on glucose minimal medium (MM) supplemented with increasing concentrations of Congo red (CR), calcofluor white (CFW), and sorbitol for 48 h. (m) Intracellular glycogen concentrations in the WT and Δ*gsyA* strains when grown in MM for 20 h. (n) Strains were grown on MM supplemented with increased concentrations of the β-glucan synthase competitive inhibitor caspofungin. Standard deviations and the average from 3 biological replicates are shown. Statistical differences were calculated using a paired *t* test (*, *P* < 0.05; **, *P* < 0.01; and ***, *P* < 0.001) based on comparison with the WT strain. Download FIG S1, PDF file, 4.0 MB. Copyright © 2018 de Assis et al. 2018 de Assis et al. This content is distributed under the terms of the Creative Commons Attribution 4.0 International license.

To assess whether PKA and the MAPKs SakA and MpkC are also involved in the regulation of hexo/glucokinase in A. fumigatus, hexo/glucokinase activities were determined in the respective protein kinase deletion mutants (Fig. 2b). Hexo/glucokinase activity was not significantly altered in the Δ*pkaC1* and Δ*sakA* Δ*mpkC* strains but was increased under some conditions in the Δ*sakA* and Δ*mpkC* single mutants compared to the WT strain (Fig. 2b). These results suggest that, in contrast to A. nidulans, PKA does not affect hexo/glucokinase activity in A. fumigatus. Furthermore, although there is some difference in hexo/glucokinase activities in the Δ*sakA* and Δ*mpkC* single mutants, it is likely not the main reason for the severe reduction in glucose consumption in these strains (Fig. 1c).

In the absence of a stress condition, the Δ*sakA* and Δ*sakA* Δ*mpkC* strains have been shown to present altered exposure of carbohydrates on the cell wall surface as well as increased sensitivity to cell wall-damaging agents (36, 41). To determine whether cell wall surface carbohydrates were also altered in the MAPK mutant strains and in the Δ*pkaC1* strain in the presence of osmotic stress, wheat germ agglutinin-fluorescein isothiocyanate (WGA-FITC) and calcofluor white (CFW) binding capacities of cell wall NAG (*N*-acetylglucosamine) and chitin, respectively, were determined. All strains (with the exception of WGA-FITC staining of the Δ*mpkC* mutant) displayed reduced exposure of NAG and chitin on the cell wall surface under both control and osmotic stress conditions (Fig. 2c and d). These results underline the importance of the HOG and PKA pathways for correct cell wall structure and/or organization under osmotic stress.

### PKA is involved in controlling intracellular glycogen and trehalose levels during osmotic stress.

**FIG 3:**
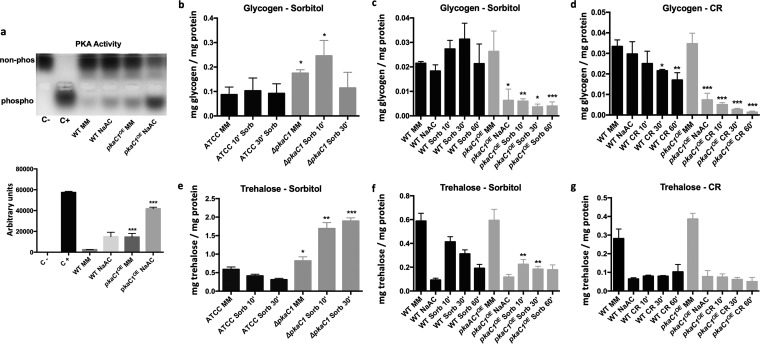
PkaC1 downregulates trehalose and glycogen levels. The wild-type (WT) and (*p*)*acuD*::*pkaC1^OE^* [*pkaC1-*overexpressing strain, regulated by the (*p*)*acuD* promoter, inducible in the presence of acetate] strains were grown in MM for 20 h, transferred to 100 mM sodium acetate (NaAC) for 2 h before (a**)** the PKA activity was measured in agarose gel and quantified (where “non-phos” represents the nonphosphorylated substrate and “phospho” the phosphorylated substrate of PKA). The WT (ATCC) and Δ*pkaC1* strains were grown in MM for 20 h before 1 M sorbitol was added, and intracellular glycogen (b) and trehalose (e) levels were measured at the indicated time points. (c and d) Glycogen content in the PKA-overexpressing strain (grown under the same conditions as specified under panel a) in the presence of (c) osmotic stress, induced by the addition of 1 M sorbitol or (d) cell wall stress, induced by the addition of 300 μg/ml Congo red (CR) for the indicated time points. (f and g) Trehalose content in the PKA-overexpressing strain (grown under the same conditions as specified under panel a) in the presence of (f) osmotic stress, induced by the addition of 1 M sorbitol, or (g) cell wall stress, induced by the addition of 300 μg/ml Congo red (CR), for the indicated time points. Standard deviations and the average of 3 biological replicates are shown. Statistical differences were calculated using a paired *t* test (*, *P* < 0.05; **, *P* < 0.01; and ***, *P* < 0.001) based on comparison with the WT strain at the same time point.

In Saccharomyces cerevisiae, the PKA pathway is responsible for the degradation of glycogen and trehalose when growing on fermentable carbon sources such as glucose, sucrose, and fructose, through inhibition of the activity of enzymes involved in glycogen and trehalose synthesis and activation of enzymes involved in the breakdown of these carbohydrates (42). To determine whether PKA exerts a similar function in A. fumigatus, intracellular glycogen and trehalose levels were measured in a strain deleted for the PKA catalytic subunit *pkaC1* under osmotic stress conditions (Fig. 3b and e). Intracellular glycogen and trehalose levels were significantly higher under all conditions in the Δ*pkaC1* strain compared to the wild-type (WT) strain. Consistently, overexpression of *pkaC1* by addition of sodium acetate [by placing the gene under the sodium acetate-inducible promoter (*p*)*acuD* (*pkaC1^OE^*)] (Fig. 3a) resulted in a reduction of intracellular glycogen and trehalose levels in the presence of osmotic stress (Fig. 3c and f). These results suggest that, like in S. cerevisiae, PKA is involved in regulating cellular glycogen and trehalose levels.

To determine whether different cell wall stresses induce a similar, PKA-dependent response in A. fumigatus, glycogen and trehalose levels were assessed in the *pkaC1^OE^* strain when mycelia were exposed to the cell wall-damaging agent Congo red (CR). In the presence of CR, cellular glycogen levels were significantly more reduced in the *pkaC1^OE^* strain than in the wild type (Fig. 3d). No change in trehalose levels was observed compared to the WT strain (Fig. 3g), as trehalose levels were already low after sodium acetate induction without a noticeable increase of trehalose levels after CR treatment. In agreement, the WT strain had reduced glycogen levels upon exposure to CR, but not when exposed to sorbitol (Fig. 3c and d), whereas both types of cell wall stresses induced a reduction in intracellular trehalose levels, albeit to different extents (Fig. 3f and g).

### Glycogen and trehalose metabolism is regulated by *creA*.

In Neurospora crassa, the carbon catabolite repressor CRE-1 has been shown to be important for glycogen metabolism under nonstress conditions (43). Similarly, S. cerevisiae Snf1p, a regulator of the CRE-1 homologue Mig1p, is responsible for glycogen accumulation in the presence of low glucose concentrations that favors a shift to respiratory metabolism (44). In A. nidulans, the PKA catalytic subunit PkaA regulates cellular localization of the carbon catabolite repressor CreA (20). These studies suggest potential communication between CreA-mediated carbon catabolite repression (CCR) and PKA-signaling in glucose utilization under different stress conditions.

**FIG 4:**
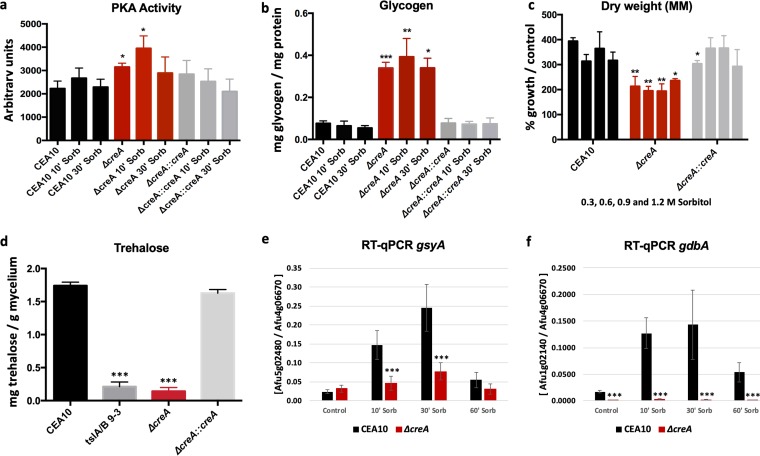
A differential role of CreA in glycogen and trehalose metabolism. Strains were grown in complete medium for 20 h before 1 M sorbitol was added, and (a) PKA activity and (b) intracellular glycogen concentrations were determined. (c) Deletion of *creA* causes a growth defect under osmotic stress conditions. Fungal dry weight was measured after strains were grown in MM supplemented with 0.3, 0.6, 0.9, and 1.2 M sorbitol for 48 h. (The percentage of growth compared to the control condition without sorbitol is considered 100%.) (d) Intracellular trehalose concentrations were reduced in the *creA* deletion strain. As a control, trehalose levels were also measured on MM in the Δ*tslA*/*B* strain carrying the deletion of two trehalose biosynthetic enzymes. (e and f) CreA regulates the expression of genes encoding enzymes involved in glycogen metabolism. RT-qPCR of the (e) glycogen synthase-encoding gene *gsyA* and (f) the glycogen debranching-encoding gene *gdbA* when strains were grown under the same conditions as specified in panel a**)**. Standard deviations and the average from 3 biological replicates are shown. Statistical differences were calculated using a paired *t* test (*, *P* < 0.05; **, *P* < 0.01; and ***, *P* < 0.001) based on comparison with the WT strain at the same time point.

PKA activity was measured in the Δ*creA* strain and found to be significantly increased under the control condition and after 10 min of exposure to 1 M sorbitol (Fig. 4a). This result suggests an interaction between PKA and CCR in A. fumigatus and implies an importance of CreA for intracellular glycogen degradation. The importance of CreA for glycogen metabolism in A. fumigatus was therefore determined. Intracellular glycogen levels were measured in the Δ*creA* strain under osmotic stress conditions. The Δ*creA* strain presented a high accumulation of intracellular glycogen levels under all conditions, which was accompanied by a reduction in fungal biomass (Fig. 4b and c). To confirm the role of CreA in glycogen metabolism during osmotic stress, the levels of expression of *gsyA*, encoding glycogen synthase, and of the glycogen debranching enzyme *gbdA* were assessed by reverse transcription-quantitative PCR (RT-qPCR). The expression of *gsyA* was reduced in the Δ*creA* strain during 10 and 30 min of exposure to sorbitol compared to the WT strain, whereas the expression of *gbdA* was severely reduced under all conditions (Fig. 4e and f). The inability of the Δ*creA* strain to efficiently break down glycogen caused this strain to use intracellular trehalose, which was confirmed by a severe reduction in intracellular trehalose levels that was similar to those observed for strains deleted in the genes *tslA* and *tslB*, which encode enzymes involved in trehalose biosynthesis (45) (Fig. 4d). Collectively, these results confirm the regulatory role of CreA in glycogen and trehalose metabolism in A. fumigatus.

### PKA and the MAPK SakA physically interact.

**FIG 5:**
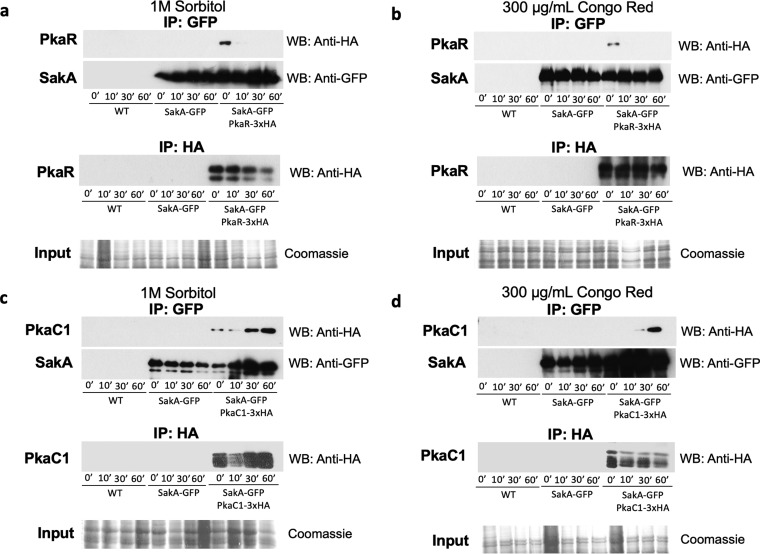
PKA and the HOG MAPK SakA physically interact under osmotic and cell wall integrity stress conditions. The wild-type (WT), SakA-GFP PkaR-3×HA, and SakA-GFP PkaC1-3×HA strains were grown in either YG or YPD for 20 h before 1 M sorbitol or 300 μg/ml Congo red (CR) was added. Total protein was extracted from mycelia, and immunoprecipitation (IP) of SakA-GFP was carried out before the Western blot was run and analyzed with anti-GFP or anti-HA antibody for the (a and b**)** PkaR-3×HA interactions with SakA-GFP and (c and d) PkaC1-3×HA interactions with SakA-GFP. IP of PkaR-3×HA or PkaC1-3×HA was carried out, and Western blots were analyzed with anti-HA antibody (lower panel). Coomassie-stained protein gels of the total protein extracts were used as a loading control.

The aforementioned results suggest cooperation between the PKA and HOG pathways. To determine whether PkaC1 and SakA physically interact under osmotic stress, the PKA regulatory (PkaR) and catalytic (PkaC1) subunits were tagged with 3× hemagglutinin (HA) in the SakA-GFP (green fluorescent protein) background strain and Western blots after immunoprecipitation (IP) were carried out. Strains were grown in complete medium for 20 h before osmotic stress was induced with 1 M sorbitol for 10, 30, and 60 min. IP was carried out for GFP, and membranes were incubated with anti-HA antibody. In the absence of osmotic stress, PkaR physically interacted with SakA, and this interaction was lost upon exposure to sorbitol (Fig. 5a). PkaC1 interacts with SakA under the control condition and also after prolonged incubation under osmotic stress conditions (Fig. 5c). A similar pattern of physical interaction between PkaR, PkaC1, and SakA was observed when A. fumigatus was exposed to the cell wall-damaging agent CR for 10, 30, and 60 min (Fig. 5b and d). In S. cerevisiae and A. nidulans, the PKA regulatory and catalytic subunits form a complex, with the dissociation of the regulatory subunit resulting in PKA activation (23, 24, 46).

In order to understand how protein phosphorylation influences the PKA response to cell wall damage, a preliminary phosphoproteomics study was performed to characterize protein phosphorylation in mycelia when grown in the presence of 200 mM CR for 10 min. We did not observe the presence of PkaC1 phosphopeptides in this data set. However, PkaR phosphopeptides (S24 in boldface) 14-ISEEEEYEVT**S**PTDPTFR-31 and (S39 in boldface) 35-DDKY**S**PIQK-43 were observed with 3.5-fold more abundance and S39 was exclusively identified in the wild-type strain upon exposure to CR (Table 1). NetPhos predicts that these two phosphopeptides are phosphorylated by p38 MAPK (http://www.cbs.dtu.dk/services/NetPhos-3.1/) (Table 1). The phosphopeptide (S24 in boldface) 14-ISEEEEYEVT**S**PTDPTFR-31 has reduced abundance in the Δ*mpkC* strain compared to the wild-type strain (upon CR, 0.47-fold) and was not identified in the Δ*sakA* and Δ*sakA* Δ*mpkC* strains upon CR exposure (Table 1). Interestingly, the phosphopeptide (S39 in boldface) 35-DDKY**S**PIQK-43, identified only in the wild type exposed to CR, is not present in any of the MAPK mutant strains (Table 1). In the Δ*mpkC* and Δ*sakA* Δ*mpkC* mutants, the phosphopeptide (S24 in boldface) 14-ISEEEEYEVT**S**PTDPTFR-31 is present only under the control condition, and it has increased abundance compared to the wild-type strain (2.83- and 27.5-fold, respectively [Table 1]). Surprisingly, we were not able to identify any PkaC1 phosphopeptide residue in the Δ*sakA* mutant (Table 1).

**TABLE 1:** Phosphopeptides identified in the wild-type and MAPK mutant upon exposure or nonexposure to Congo red

Condition	PR^*a*^	Phosphopeptide	Abundance					Ratio	*P* value	p38 MAPK activity (AU)^*c*^
			WT		Δ*mpkC*		*T*_0_ Δ*sakA* Δ*mpkC*			
			*T*_0_^*b*^	CR	CR	*T*_0_				
CR										
WT	S24	ISEEEEYEVT**S**PTDPTFR	3e+07	1.1e+08	NI^*d*^	NI	NI	WT+CR/*T*_0_ WT = 3.37	0.009	0.484
	S39	DDKY**S**PIQK	NI	1.9 + 06	NI	NI	NI	Identified only with CR	0.02	0.532
Δ*mpkC*	S24	ISEEEEYEVT**S**PTDPTFR	NI	1.1e+08	4.5e+07	NI	NI	Δ*mpkC*+CR/WT+ CR = 0.41	0.03	0.484

*T*_0_										
Δ*mpkC*	S24	ISEEEEYEVT**S**PTDPTFR	3e+07	NI	NI	9.2 + 07	NI	*T*_0_ Δ*mpkC*/*T*_0_ WT = 2.83	0.04	0.484
Δ*sakA* Δ*mpkC*	S24	ISEEEEYEVT**S**PTDPTFR	1e+06	NI	NI	NI	3e+07	*T*_0_ Δ*sakA* Δ*mpkC*/*T*_0_ WT = 27.5	0.002	0.484

a PR, phosphoresidue.

b *T*_0_, time zero.

c Predicted by NetPhos (http://www.cbs.dtu.dk/services/NetPhos-3.1/).

d NI, not identified.

The aforementioned results indicate that SakA interacts with PkaR and PkaC1, resulting in further PKA activation. This activation probably occurs through PkaR as two phosphopeptides were observed only for the PkaR subunit, with one of them not being present in the MAPK mutant strains in the presence of CR-induced cell wall damage.

## DISCUSSION

Diverse intracellular signaling pathways allow fungi to quickly adapt to environmental stimuli. Often, substantial cross talk exists between these signaling pathways, governed by either direct or indirect interactions between pathway-specific proteins (37, 47, 48). Mitogen-activated protein kinase (MAPK) pathways respond to extracellular cues and transmit this information intracellularly, therefore regulating different cellular processes, including various stress responses, such as fungal cell wall damage and cell proliferation and differentiation (49). The genome of A. fumigatus encodes four MAPKs: (i) MpkA, the central regulator of the cell wall integrity (CWI) pathway that responds to drug-induced cell wall damage and reactive oxygen species; (ii) MpkB, which remains largely uncharacterized; and (iii) MpkC and SakA, the main effectors of the HOG (high-osmolarity glycerol) pathway with roles in multiple stress tolerance (19, 50).

Recently, we demonstrated that the HOG MAP kinases SakA and MpkC are essential for the response to cell wall damage (36). The Δ*sakA* and double Δ*mpkC* Δ*sakA* mutants were more sensitive to osmotic and oxidative stresses and to cell wall-damaging agents. Both MpkC-GFP and SakA-GFP translocated to the nucleus upon osmotic stress and cell wall damage, with SakA-GFP showing a quicker response (36). The phosphorylation state of MpkA was determined postexposure to high concentrations of CR and sorbitol. In the wild-type strain, MpkA phosphorylation levels progressively increased in both treatments. In contrast, the Δ*sakA* mutant had reduced MpkA phosphorylation, and surprisingly, the double Δ*mpkC* Δ*sakA* mutant had no detectable MpkA phosphorylation (36). Based on these previous observations and the results observed in this article, we propose that both the CWI and HOG pathways collaborate and that MpkC could act by modulating SakA activity upon exposure to several types of stresses and mobilization of storage sugars during cell wall biosynthesis. This hypothesis is corroborated by the fact that A. nidulans SakA and MpkC not only physically interact, but also show opposite and common functions during stress responses and development (51, 52).

Despite in-depth characterization of the CWI and HOG MAPK effectors, pathway cross talk and further intracellular signaling events remain largely unknown in how A. fumigatus responds to cell wall damage. An understanding of these signaling events and the response in A. fumigatus is important since the fungal cell wall and the accompanying CWI are a crucial determinant for fungal pathogenesis and virulence (53). Therefore, we were specifically interested in the connection between the activation of intracellular glycogen and trehalose storage compounds and CWI pathways, since the large majority of cell wall polysaccharides are biosynthesized from the monosaccharide glucose, which can be derived from intracellular glycogen and trehalose storage compounds (10, 54, 55).

This study contributes to the understanding of the complex mechanism underlying glycogen and trehalose utilization during osmotic and cell wall stresses and implicates cooperation between the PKA and HOG pathways as master regulators of this process. Similarly to other fungi (21, 56–59), the PKA catalytic subunit PkaC1 was shown to be required for regulation of intracellular glycogen and trehalose levels. This regulation may be indirect and acting through the carbon catabolite repressor (CCR) protein CreA, which was shown to be crucial for maintenance of intracellular glycogen and trehalose levels under control and stress conditions. In A. nidulans, the PkaA catalytic subunit is controlling CCR via CreA, resulting in altered secretion of biomass-degrading enzymes such as xylanases and cellulases (20). CreA is a transcription factor that controls the expression of the glycogen synthase *gsyA* and the glycogen debranching enzyme *gbdA*-encoding genes (Fig. 4e and f). Whether CreA binds directly to the promoter region of these genes remains to be determined, but preliminary *in silico* analysis found a putative CreA binding site (5′-GCGGGG-3′; the consensus binding sequence for CreA is 5′-SYGGRG-3′) in the 5′ untranscribed region (UTR) of *gdbA* at −279 bp from the start codon, suggesting that CreA may bind to some genes encoding enzymes required for glycogen metabolism. Moreover, the inability of the Δ*creA* strain to break down glycogen causes depletion of intracellular trehalose (Fig. 4d), which could be the reason for the increased sensitivity of this strain to cell wall-perturbing agents (60). The regulation of glycogen and trehalose genes by *creA* was previously observed in A. fumigatus, where the deletion of *creA* promotes repression of genes involved in trehalose and glycogen metabolism (60). In addition, PKA activity was deregulated in the *creA* deletion strain. Reduced intracellular trehalose content was also observed in the A. nidulans loss-of-function *creA*^d30^ mutant (61). Future work that studies posttranslational modifications, such as phosphorylation on the respective proteins, will result in describing the mechanism behind CreA and PKA interaction and a better understanding of the control of carbohydrate metabolism.

**FIG 6:**
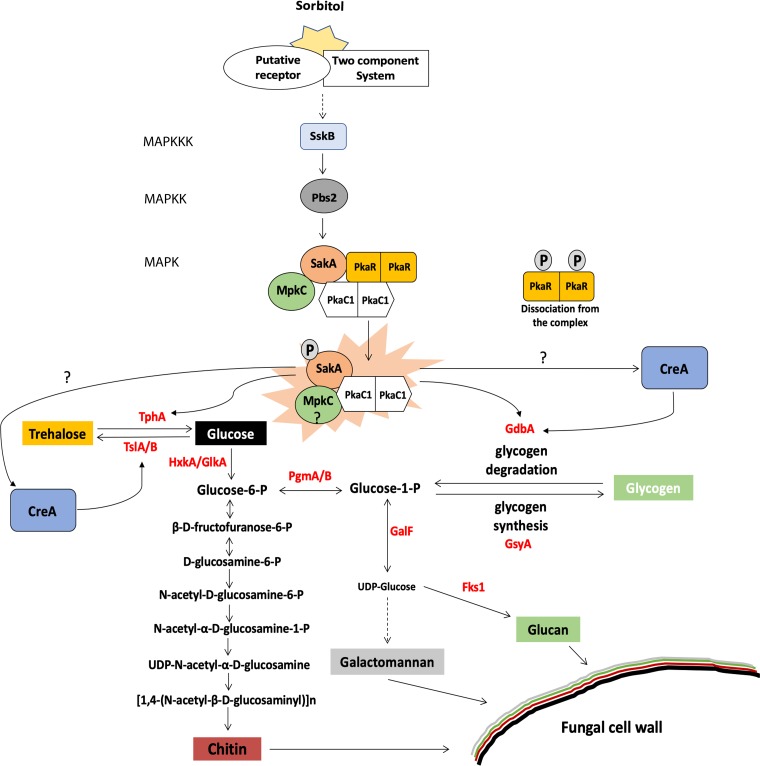
The HOG and PKA pathways collaborate to regulate glycogen and trehalose metabolism for cell wall polysaccharide precursor biosynthesis during osmotic stress conditions. In the absence of osmotic stress, the PkaR and PkaC1 subunits form a protein complex with the HOG MAPK kinases SakA and MpkC. Upon the detection of osmotic stress, the HOG MAPK cascade is activated, signaling to Pbs2, which in turn phosphorylates SakA. The active SakA acts upon the PKA regulatory subunit, resulting in the dissociation of PkaR from the complex and the activation of PkaC1. The MpkC-SakA-PkaC1 complex can now control trehalose and glycogen metabolism to counteract the osmotic stress via two distinct pathways: (a) indirectly, through controlling the transcription factor of the carbon catabolite repression CreA that regulates the expression of genes encoding enzymes required for glycogen and trehalose utilization; and (b) directly, through controlling the activity of enzymes involved in glycogen and trehalose metabolism. The degradation of glycogen and trehalose results in the release of glucose, serving as a precursor for chitin, glucan, and galactomannan biosynthesis, through a variety of enzymatic steps. The chitin and galactomannan biosynthetic pathways were predicted based on homology with Saccharomyces cerevisiae using the BioCyc Pathway/Genome Database Collection website. Dashed arrows depict several steps in the pathway. TphA, trehalase phosphorylase; TslA/B, trehalose 6-phosphate synthase; HxkA, hexokinase; GlkA, glucokinase; PgmA/B, phosphoglucomutase; GalF, UTP-glucose-1-phosphate uridylyltransferase; Fks1, β-1,3-glucan synthase; GdbA, glycogen debranching enzyme; and GsyA, glycogen synthase.

A previous study (39) suggested that the HOG MAPKs SakA and MpkC are also involved in maintaining intracellular glycogen and trehalose levels, and this was confirmed in this work, with SakA being especially important for this process. Furthermore, SakA and MpkC are also required for correct glucose uptake and subsequent intracellular glucose signaling, as shown by a significant reduction in PKA activity in the Δ*sakA* and Δ*sakA* Δ*mpkC* strains under all tested conditions. Alternatively, a defect in PKA activity may be the direct cause for the observed glucose uptake profile in these strains, as has previously been reported for A. nidulans (20). Nevertheless, SakA physically interacted with MpkC (51) and also the PKA regulatory subunit under control conditions and with the PKA catalytic subunit under stress conditions, confirming the communication between the HOG and PKA pathways. This led us to propose as a hypothesis, a mechanism whereby SakA, MpkC, PkaR, and PkaC1 form a complex under control conditions. Upon the detection of extracellular osmotic stress, the HOG pathway is activated (62, 63), resulting in SakA phosphorylation (36), which subsequently causes the dissociation of the PkaR regulatory subunit, probably as a result of SakA-mediated phosphorylation, from PkaC1 (Fig. 6). The PKA complex is now active and can phosphorylate enzymes and regulators (e.g., CreA) required for trehalose and glycogen degradation. This hypothesis is in agreement with previously described mechanisms of PKA regulation and activation in Aspergillus and other fungi (24, 26, 44). We performed a preliminary investigation into the phosphoproteome of the wild-type and MAPK mutant strains under CR exposure and identified two PkaR phosphopeptides present in the wild-type strain. However, the differential abundance of them in the mutant strains suggested these two phosphopeptides can be involved in the observed cooperation between PKA activation and MAPKs. Shwab et al. identified the same two A. fumigatus phosphopeptides conserved in A. nidulans, S. cerevisiae, Schizosaccharomyces pombe, C. neoformans, and Mucor circinelloides and demonstrated that they are downregulated in the presence of the noncompetitive β-1,3-glucan synthase inhibitor caspofungin (64). These authors also constructed a strain in which both phosphorylation sites were mutated (PkaR^S24A S39A^) and observed that this strain had reduced radial growth (64). The exact mechanism underlying PKA activity regulation and phosphorylation in the presence of different environmental cues remains subject to further investigation.

Disturbances in the aforementioned signaling pathways resulted in altered fungal cell wall composition, as shown by a reduction in cell wall chitin in the HOG MAPK and PKA deletion strains. Furthermore, the Δ*creA* strain has also been shown to be highly sensitive to cell wall-damaging agents (60), further supporting a role for this transcription factor in regulating intracellular glucose metabolism that is crucial for generating cell wall polysaccharide precursors. The observed cell wall-associated differences are likely to be the result of metabolic disturbances in the pathways that generate cell wall polysaccharide precursors. How this is achieved and whether it occurs at a transcriptional or posttranscriptional level remains to be determined.

Similarly, deletion of genes encoding the glycolysis-associated hexo/glucokinases HxkA and GlkA resulted in altered fungal cell wall composition and/or structure, as shown by a severe sensitivity of these strains to fungal cell wall-damaging agents such as CR and CFW, as well as to osmotic stress. These enzymes are crucial for A. fumigatus virulence, as the Δ*hxkA* Δ*glkA* strain was severely attenuated in two different murine models for invasive aspergillosis (Fig. S1h and k). In A. fumigatus, the metabolism of fructose, released after the degradation of sorbitol, was shown to be dependent on hexokinase, further highlighting the importance of this enzyme for sugar utilization in Aspergillus spp. (18, 65).

In addition, the biosynthesis of fungal cell wall polysaccharides may also be regulated in a condition-specific manner. Although the deletion of the glycogen synthase-encoding gene *gsyA* completely abolished glycogen synthesis (Fig. S1m), it did not result in increased sensitivity to osmotic stress or CR-induced cell wall disturbance (Fig. S1l). However, such a mutant lost the caspofungin paradoxical effect (CPE) (Fig. S1n). The CPE describes increased caspofungin resistance when significantly exceeding the MIC of the drug. Prolonged exposure to caspofungin results in an increase in cell wall chitin (66), ultimately indicating a deficiency in recovering from partial depletion of β-1,3-glucan when glycogen is not available. In agreement, the Δ*pkaC1* strain showed alterations in growth during the CPE, but only in liquid medium and not on solid medium (67). Furthermore, this study showed that various types of cell wall stressors result in different intracellular responses, as well as in strain-specific differences, further underlining the complexity of these signaling events. The A. fumigatus Afs35 strain responds to osmotic stress by breaking down glycogen instead of trehalose, whereas the ATCC strain uses up intracellular trehalose instead of glycogen under the same conditions (Fig. 1 and 3). Indeed, heterogeneity in clinical isolates of A. fumigatus in an infection-related context has been described before (68).

In summary, this study describes the communication between the HOG MAPK and PKA pathways that ultimately regulate glycogen and trehalose metabolism, which is crucial for cell wall polysaccharide precursor availability. The HOG MAPK pathway is activated upon osmotic stress, thereby taking over the role of a sensor, whereas PKA mainly regulates downstream signals by targeting transcription factors such as CreA (Fig. 6). Disturbances in these pathways through the deletion of their main effector proteins (e.g., SakA and PkaC1) impact cell wall composition and/or structure and result in attenuated virulence. The importance of glycogen for filamentous fungal pathogenesis has been reported previously in plant pathogens (30). Similarly, trehalose is important for germination and for establishing an infection (28, 29, 69, 70). In conclusion, this study reports on the mechanistic signaling and regulation events underlying A. fumigatus cell wall polysaccharide mobilization, which is critical for fungus-host interactions. Further elucidation of these mechanisms may yield new insights into improving/discovering cell wall-targeting antifungal drugs.

## MATERIALS AND METHODS

### Strains, media, and growth conditions.

All A. fumigatus strains used in this study are listed in Text S1 in the supplemental material. This study used an array of strains from different backgrounds (Text S1), and all results with these strains were compared to the corresponding wild-type strain. In addition, there are no substantial phenotypic differences among the background strains CEA17, ATCC 46645, and Afs35 that would interfere with the interpretation of the results. Strains were grown in either complete medium (YG [2% wt/vol glucose, 0.5% wt/vol yeast extract, 1 ml trace elements] [71] or YPD [1% wt/vol yeast extract, 2% wt/vol glucose, 2% wt/vol peptone]) or minimal medium (MM [1% wt/vol glucose or 1% wt/vol acetate, 50 ml salt solution, 1 ml trace elements, pH 6.5]) as previously described (20). Throughout this study, strains were incubated at 37°C.

10.1128/mBio.01952-18.1 TEXT S1 Supplementary methods and strains. Download Text S1, DOCX file, 0.01 MB. Copyright © 2018 de Assis et al. 2018 de Assis et al. This content is distributed under the terms of the Creative Commons Attribution 4.0 International license.

### Enzymatic assays.

Intracellular trehalose levels (Megazyme) and PKA activity (Promega) were carried out according to the manufacturer’s instructions. Intracellular glycogen levels and extracellular glucose concentrations were quantified as described previously (72). Fungal cell wall surface carbohydrates were measured as previously described (36, 41). Hexo/glucokinases activities were assessed as described in reference 20.

### Coimmunoprecipitation assay.

Proteins were extracted from mycelia grown under the specified conditions by being ground to a fine powder under liquid nitrogen and resuspended in buffer B250 as described by Assis et al. (73). Samples were incubated with 20 μl of anti-HA antibody H3663-200UL (Sigma)-loaded magnetic beads or with 20 μl GFP-Trap (Chromotek) resin for 4 h on a horizontal shaker. Magnetic beads were washed two times with buffer B250 and once with protein extraction buffer (73) before samples were incubated with 20 μl SDS-sample buffer and incubated at 95˚C for 5 min. Samples were run on a 12% SDS-PAGE gel, and Western blotting was carried out as described previously (20).

### RT-qPCR.

RNA extraction, cDNA synthesis, and RT-qPCR were carried out as described previously (71). Gene expressions were normalized with gene Afu4g0660 as this gene is not modulated under the control and sorbitol-rich conditions, as shown by RNA-seq (39) and confirmed by RT-qPCR.
