# Supplementary figures and images for: Protein Kinase A and High-Osmolarity Glycerol Response Pathways Cooperatively Control Cell Wall Carbohydrate Mobilization in Aspergillus fumigatus
Source: mBio. 2018 Dec 11;9(6):e01952-18. doi: 10.1128/mBio.01952-18 (PMC6299480; doi:10.1128/mBio.01952-18)

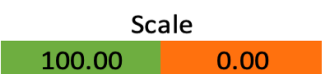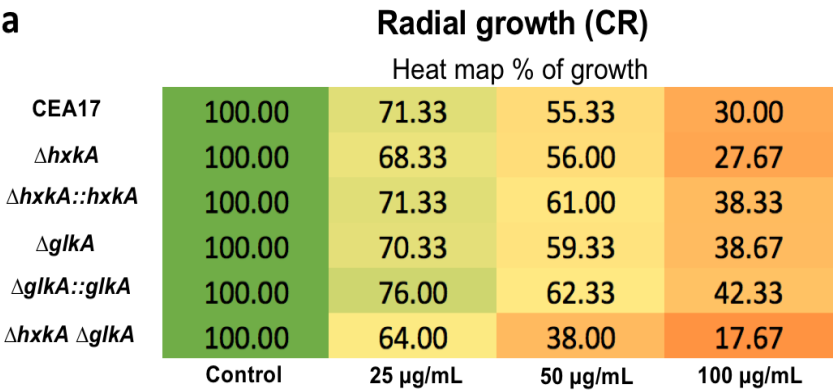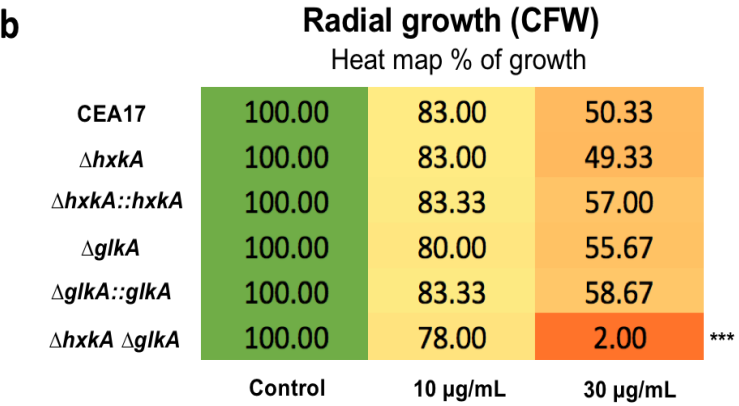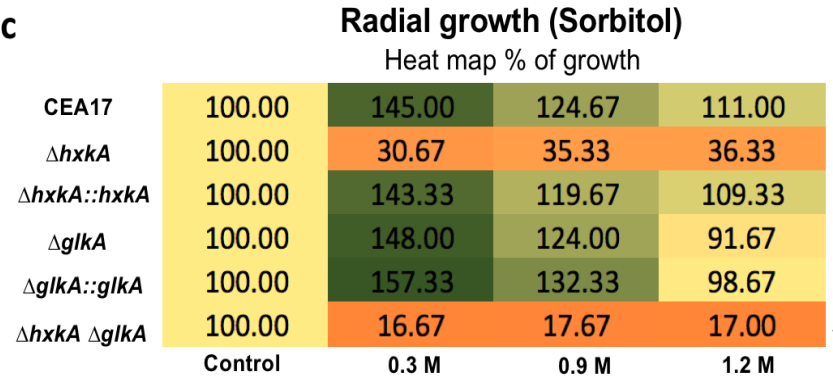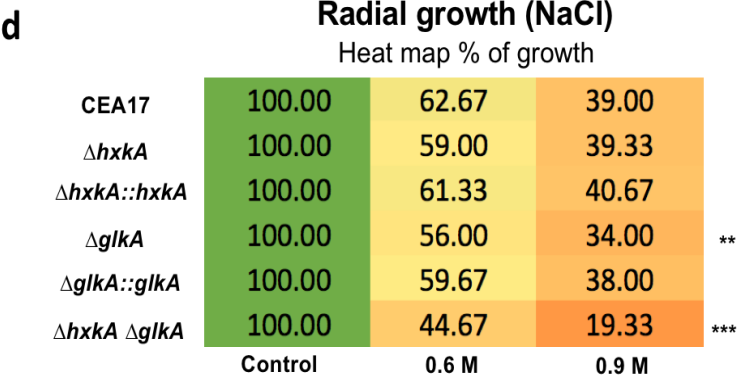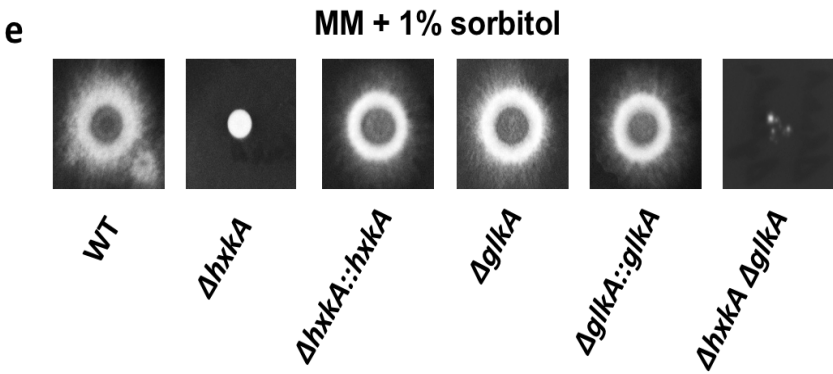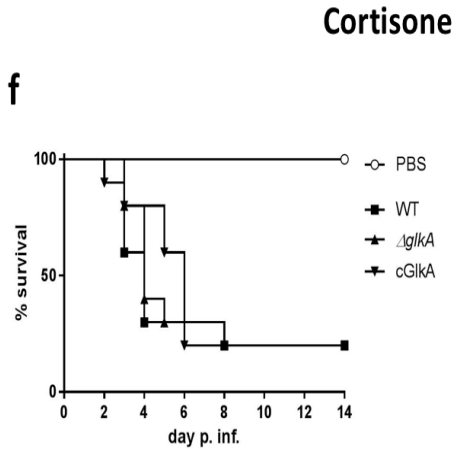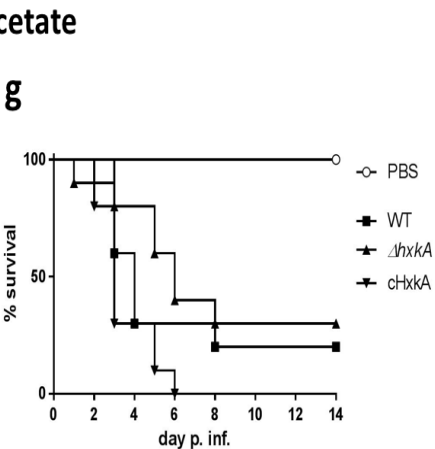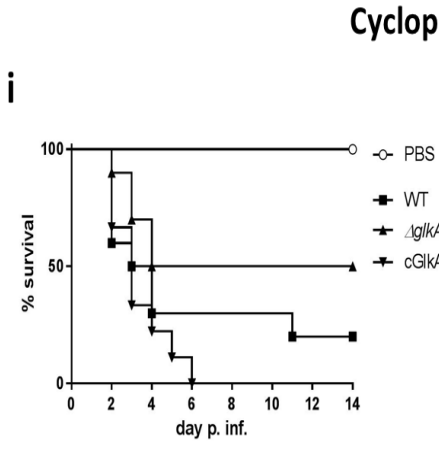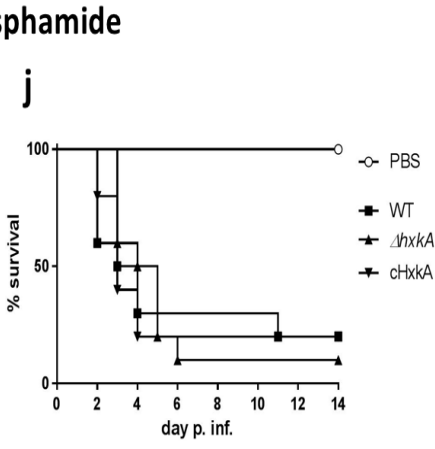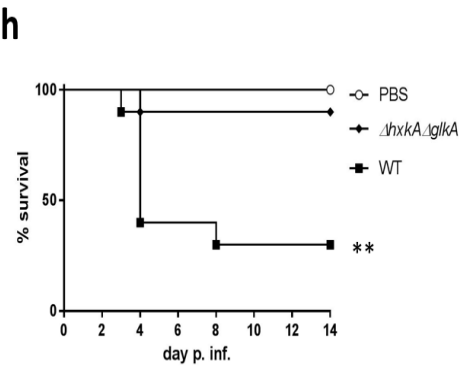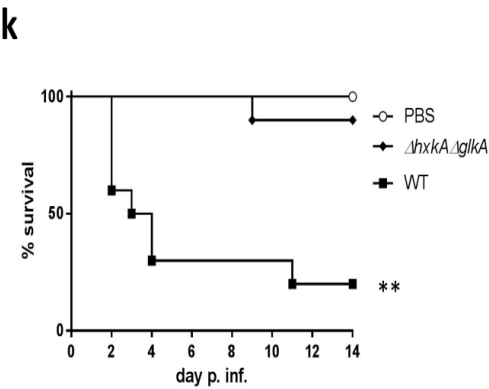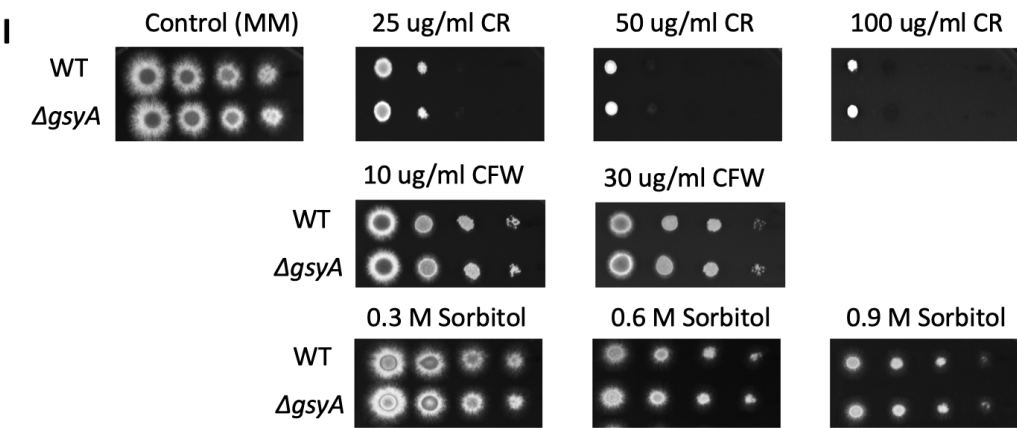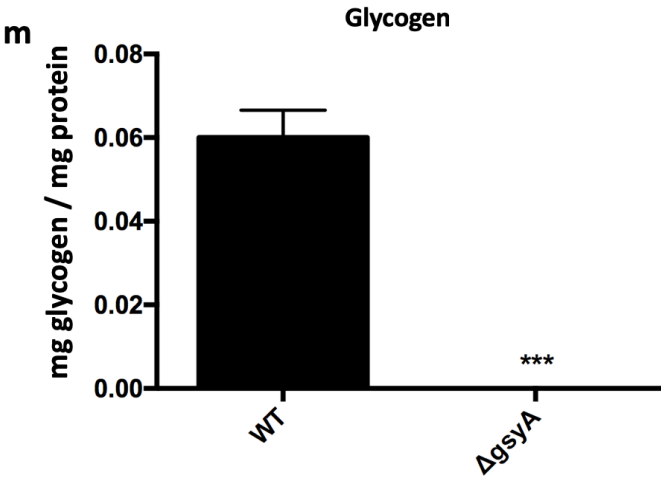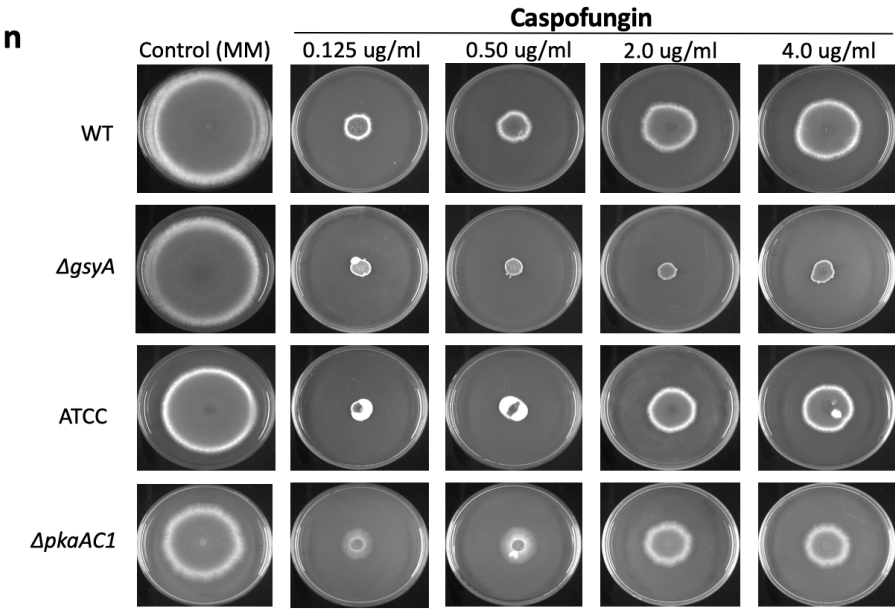

Supplement: FIG S1 [file mbo006184212sf1.pdf]
