# Supplementary material for: Protein Kinase A and High-Osmolarity Glycerol Response Pathways Cooperatively Control Cell Wall Carbohydrate Mobilization in Aspergillus fumigatus
Source: mBio. 2018 Dec 11;9(6):e01952-18. doi: 10.1128/mBio.01952-18 (PMC6299480; doi:10.1128/mBio.01952-18)
Supplement: TEXT S1 [file mbo006184212s1.docx]

**Protein kinase A (PKA) and high osmolarity glycerol (HOG) response pathways cooperatively control cell wall carbohydrate mobilization in *Aspergillus fumigatus***

Leandro José de Assis^1^, Adriana Manfiolli^1^, Eliciane Mattos^1^, Iran Malavazi^2^, Ilse D. Jacobsen^3^, Matthias Brock^4^, Robert A. Cramer^5^, Arsa Thammahong^6^, Daisuke Hagiwara^7^, Laure Nicolas Annick Ries^8^, and Gustavo Henrique Goldman^1^.

**Supplementary Methods section**

**Ethics statement**

Animal studies were conducted in accordance with the recommendations of the European Community and were approved by the Animal Care and Usage Committee of the Landesamt Thüringen, Bad Langensalza, Germany (permission no. 03-001/08).

**Murine models for aspergillosis**

Virulence of *A. fumigatus* strains was analyzed in both a corticosteroid model (using outbred female CD-1 mice) and leucopenic mouse model (using BALB/c mice) for pulmonary invasive aspergillosis (72, 73) 6 to 8 weeks old mice were obtained from Charles River Laboratories Germany, kept under specific pathogen-free conditions in groups of five in isolated ventilated cages with sterile food and water provided *ad libitum*. Leucopenia was achieved by intraperitoneal application of 150 mg cyclophosphamide (Sigma-Aldrich)/kg on days -4, -1, and 2, prior to and after infection on day 0, followed by application of 120 mg cyclophosphamide/kg on days 5, 8, and 11. Additionally, a single dose of cortisone acetate (200 mg/kg; Sigma-Aldrich) was injected subcutaneously on day -1. For the corticosteroid model, 25 mg cortisone acetate (Sigma Aldrich) in 200 µL PBS were injected intraperitoneally on day -3 and day 0. On day 0, intranasal infection was performed under general anesthesia (0.05 mg/kg fentanyl, 0.5 mg/kg medetomidin, and 5 mg/kg midazolam) by applying 2-4×10^5^ conidia (corticosteroid model) or 1-2×10^5^ conidia in 20 µL sterile phosphate-buffered saline (PBS) onto the nostrils. For the Δ*glkA*/Δ*hxkA* double mutant the infectious dose was doubled (4 × 10^5^ for the corticosteroid, and 2 × 10^5^ for the cyclophosphamide model) to compensate for a general reduced viability of conidia of this strain (15). Anesthesia was terminated by subcutaneous application of antidote (1.2 mg/kg naloxone, 2.5 mg/kg atipamezol, and 0.5 mg/kg flumazenil). Mice were monitored at least twice daily for development of clinical symptoms and humanely sacrificed if they met predefined humane endpoints (≥ 25% weight loss, severe lethargy, severe dyspnea, inability to move coordinately) or at the end of the experiment. Ten mice were infected per group and experiment. Statistical analysis was performed by comparing survival groups infected with each mutant strain to the respective wild type group using Kaplan Meier curves and log rank test.

**Fungal growth, protein extraction, digestion and LC-MS/MS**

About 1 x 10^7^*Aspergillus fumigatus* conidia from the wild-type (WT) and mutant strains (Δ*mpkC*, Δ*sakA*, and Δ*mpkC* Δ*sakA*) were inoculated in YG medium (0.5% Yeast extract; 1% dextrose; 0.1% trace elements) and incubated for 16h under 200 rpm rotation and 37^o^C. Subsequently, 300μg/ml of Congo Red (CR) were added and incubated for 10 min (or time zero, as control). Mycelia were then filtered using a vacuum system and frozen by liquid nitrogen. Frozen mycelia were macerated and protein extracted by the addition of TNE buffer (50 mM tris-HCl (pH 7.5), 140 mM NaCl, 5 mM EDTA, 100 mM NaF, 1 mM Na_3_VO_4_, 0.05mM *sodium β-glicerophosphate* e *EDTA-free protease inhibitor cocktail* (Roche) and 0.1 mM PMSF)) and incubation for 15 min under agitation following centrifugation at 10,000 x g for 20 min. The supernatant was collected and total protein quantified by Bradford assay. Five-hundred μg of total protein were precipitated by the addition of 20% TCA and incubated for 30 min on ice. Samples were centrifuged at 14,000 x *g* for 15 min and the supernatants were discarded. The pellet was washed three times with cold Acetone with centrifugations at 14,000 x *g,* at 4^o^C for 10 min each. Protein pellets were reduced by the addition of DTT (DL-Dithiothreitol) and alkylated by the addition of iodoacetamide. Protein digestions were performed by the addition of *Sequencing-grade Trypsin* (Promega) (1:50) for 16h at 37^o^C. Phosphopeptides were enriched by Pierce™ TiO2 Phosphopeptide Enrichment and Clean-up Kit (Thermo Fisher Scientific) according to the manufacturer’s instructions. Phosphopeptide identification and analysis were performed with at least 3 replicates on a nanoLC (NanoAcquity; Waters Corporation, Milford, MA) coupled to a Orbitrap Fusion™ Tribrid™ mass spectrometer (Thermo Fisher, San Jose, CA). Raw mass spectrometric data were analyzed in the MaxQuant environment. The MS/MS spectra were matched against *Aspergillus fumigatus* Uniprot FASTA database.
